# Construct validity and reliability of the Finnish version of the Knee Injury and Osteoarthritis Outcome Score

**DOI:** 10.1186/s12891-018-2078-7

**Published:** 2018-05-22

**Authors:** Juhani Multanen, Mikko Honkanen, Arja Häkkinen, Ilkka Kiviranta

**Affiliations:** 1 Department of Physical Medicine and Rehabilitation, Central Finland Central Hospital, Keskussairaalantie 19, FI-40620 Jyväskylä, Finland; 2 Department of Surgery, Mikkeli Central Hospital, Mikkeli, Finland; 3 Faculty of Sport and Health Sciences, University of Jyväskylä, Jyväskylä, Finland; 4 Department of Orthopaedics and Traumatology, University of Helsinki, and Helsinki University Hospital, Helsinki, Finland

**Keywords:** KOOS, Knee injury, Knee osteoarthritis, Finnish, Reliability, Validity

## Abstract

**Background:**

The Knee Injury and Osteoarthritis Outcome Score (KOOS) is a commonly used knee assessment and outcome tool in both clinical work and research. However, it has not been formally translated and validated in Finnish. The purpose of this study was to translate and culturally adapt the KOOS questionnaire into Finnish and to determine its validity and reliability among Finnish middle-aged patients with knee injuries.

**Methods:**

KOOS was translated and culturally adapted from English into Finnish. Subsequently, 59 patients with knee injuries completed the Finnish version of KOOS, Western Ontario and McMaster Osteoarthritis Index (WOMAC), Short-Form 36 Health Survey (SF-36) and Numeric Pain Rating Scale (Pain-NRS). The same KOOS questionnaire was re-administered 2 weeks later. Psychometric assessment of the Finnish KOOS was performed by testing its construct validity and reliability by using internal consistency, test-retest reliability and measurement error. The floor and ceiling effects were also examined.

**Results:**

The cross-cultural adaptation revealed only minor cultural differences and was well received by the patients. For construct validity, high to moderate Spearman’s Correlation Coefficients were found between the KOOS subscales and the WOMAC, SF-36, and Pain-NRS subscales. The Cronbach’s alpha was from 0.79 to 0.96 for all subscales indicating acceptable internal consistency. The test-retest reliability was good to excellent, with Intraclass Correlation Coefficients ranging from 0.73 to 0.86 for all KOOS subscales. The minimal detectable change ranged from 17 to 34 on an individual level and from 2 to 4 on a group level. No floor or ceiling effects were observed.

**Conclusion:**

This study yielded an appropriately translated and culturally adapted Finnish version of KOOS which demonstrated good validity and reliability. Our data indicate that the Finnish version of KOOS is suitable for assessment of the knee status of Finnish patients with different knee complaints. Further studies are needed to evaluate the predictive ability of KOOS in the Finnish population.

**Electronic supplementary material:**

The online version of this article (10.1186/s12891-018-2078-7) contains supplementary material, which is available to authorized users.

## Background

Knee pain is common complaint affecting people of all ages. In adult populations, the reported prevalence of knee pain has been 25–28% depending on the age of the examined persons and the level of pain chronicity [1–3]. In the younger age groups, knee pain is commonly secondary to increased activity, injury or contact sports [4–6]. In older people, however, knee pain that gets progressively worse over time is often sign of osteoarthritis (OA) [7, 8]. Knee OA in particular is associated with severe disability, owing to the weight-bearing function of the knee and the large range of movements it performs.

To be able to detect and treat individuals with a variety of knee problems effectively requires reliable and valid outcome measures, preferably at low cost. During recent decades, a few well-validated outcome measures have been developed for the assessment of symptoms and function in subjects with knee or hip OA [9–11]. In the elderly population, the Western Ontario and McMaster Osteoarthritis Index (WOMAC) is the self-administered instrument most commonly used to measure pain, stiffness and function in daily living. However, in younger and/or more physically active subjects, joint injuries cause knee problems more often than primary knee OA per se. Younger patients also often have higher expectations regarding physical functioning. Thus, the WOMAC may not be appropriate for these subjects. Partly for this reason, the Knee injury and Osteoarthritis Outcome Score (KOOS) was developed in the late 1990s as an extension of the WOMAC index to address problems associated with knee injuries and/or knee OA [12].

KOOS is a disease-specific, patient-reported outcome (PRO) measure assessing perceived pain, other symptoms, activities of daily living, sport and recreation functions, and knee-related quality of life. It is freely accessible, and intended for use over the short- and long-term for both research and clinical purposes. KOOS has been found to be a valid, reliable and responsive outcome measure in different patient populations with varying knee injuries (of menisci, ACL or cartilage) [13, 14], knee OA [15–19] and total knee replacement [20]. Currently, KOOS is available in 50 different languages and language variants [21]. However, the validity and reliability of the Finnish version of KOOS in subjects with different knee problems or OA have not been previously reported. Hence, the purpose of this study was to produce a Finnish version of KOOS and to evaluate its construct validity and reliability in a sample of middle-aged patients with knee injuries.

## Methods

### Cross-cultural adaptation

Prior to the implementation of the KOOS questionnaire, a cross-cultural adaptation of the measure was performed in accordance with the recommendations by Beaton et al. (2000) [22]. The American-English KOOS [12] was translated into Finnish by two translators independently, one translator was an experienced orthopedic surgeon (T1) and the other a professional translator with no medical background or special knowledge of the concepts in question (T2). Both versions were then collated in a consensus meeting. This consensus version was translated back into English independently by two Finnish-speaking translators of English origin (BT1 and BT2) unfamiliar with the original questionnaire or concepts therein. The translations into Finnish and back translations into English were thereafter discussed and collated in a second consensus meeting. This version was then pre-tested with 16 postmenopausal women with mild knee OA to confirm if all the items in the questionnaire were understandable and whether the subjects experienced problems in answering any of them.

### Patients and data acquisition

The study population comprised patients with diverse knee problems, including OA, post-traumatic injuries, meniscus and ligament problems. The patients had been referred to the Department of Orthopedics and Traumatology in Helsinki University Hospital from primary health care centers in the Hospital District of Helsinki and Uusimaa, where, based on clinical and radiographic or Magnetic Resonance Imaging findings, they were diagnosed as having OA or Anterior Cruciate Ligament (ACL), meniscus or combined ACL and meniscus injuries. For this study, all the assessed patients had to be in a clinically stable condition and not expected to undergo urgent surgery. The patients were recruited between March 2014 and May 2015 using a systematic sampling technique. The inclusion criteria were age 18 years or over, unresponsive to conservative treatment, and the ability to communicate in written Finnish. Eligible patients were informed about the study, and indicated their willingness to participate by giving their informed consent. For test-retest purposes, the participants were asked to complete KOOS twice: first, during the hospital visit, and then again 2 weeks later at home. During the hospital visit the patients were asked to fill in the KOOS along with the WOMAC, the SF-36 and the Numeric Rating Scale of Pain forms. One hundred thirty-one patients completed all four questionnaires during the hospital visit. Of these patients, 59 returned the KOOS questionnaire 2 weeks later from home by regular mail in a pre-paid envelope, yielding a sample of 59 patients for the study. The local Medical Ethics Committee of Helsinki University Hospital approved the study plan (Approval number 37/13/03/02/2014). Written informed consents were obtained and participants’ rights protected.

### Questionnaires

#### KOOS

KOOS is a patient-administered knee-specific questionnaire comprising five subscales: Pain (nine items); Symptoms (seven items); Activities of Daily Living (ADL) (17 items); Sport and Recreation (five items); and Knee-Related Quality of Life (QOL) (four items). Each item is scored from 0 (best) to 4 (worst) using a Likert type scale with 5 boxes. The raw score for each subscale is the sum of the item scores. Scores are then transformed to a 0 to 100 scale. The scores of the five subscales can be expressed as an outcome profile, higher scores indicating fewer problems. A total score has not been validated and is not recommended according to the KOOS Users Guide [21]. When filling the questionnaire the subjects are instructed to consider the previous week when answering the questions. The KOOS has a self-explanatory format and the questionnaire takes about 10 min to complete [12].

#### WOMAC

The Western Ontario and McMaster Osteoarthritis Index (WOMAC) is a disease-specific, self-administered health status instrument assessing pain, stiffness, and function in subjects with OA of the hip or knee [10]. The index consists of 24 questions in three dimensions: knee pain (5 questions), joint stiffness (2 questions) and functional ability (17 questions). These dimensions are often analyzed and reported separately, but the WOMAC index can also be aggregated into a single score. The original WOMAC is available in two formats: Visual Analog Scales (VAS) and Likert-boxes. In this study, we used the VAS format (0–100 mm), where the sum of the raw scores was transformed to a 0–100 scale. Higher scores indicate a higher level of joint pain, joint stiffness and functional limitation. For this study, however, to allow comparison between the KOOS and WOMAC outcomes, we reversed the scoring direction of the WOMAC outcomes, meaning that higher scores indicate decreased pain, joint stiffness and functional limitation. WOMAC has been extensively tested for reliability, validity and responsiveness in different countries [23]. The Finnish version of WOMAC has been validated for the short- and long-term follow-up of patients scheduled for total knee or hip arthroplasty [24].

#### SF-36

Health-related quality of life was measured using the Short-Form 36 Health Survey (SF-36) [25]. SF-36 is a generic survey comprising 8 distinct dimensions of health status: Physical Functioning, General Health, Vitality, Mental Health, Role- Physical, Role-Emotional, Social Functioning and Bodily Pain. The scale runs from 0 to 100 in each dimension, with a higher score indicating better health.

#### Pain-NRS

The subjective intensity of pain in general and in different body regions was measured on the numeric pain rating scale (Pain-NRS). The body regions in this study were neck, back, upper limb, lower limb and knee. The Pain-NRS is an 11-point scale ranging from 0 to 10, where 0 represents “no pain” and 10 represents “the most intense pain imaginable”. For this study, however, as with WOMAC we reversed the scoring direction of the Pain-NRS outcomes, meaning that higher scores indicate “no pain” and lower scores indicate “the most intense pain imaginable”. When filling the form, patients are asked to select the value that best describes the intensity of pain that they have experienced during the past week.

#### Background data

During their visit to the outpatient clinic, in addition to demographic data, patients were asked for anamnestic information regarding their knee problem, and to describe the intensity of their habitual physical activity (low = 1, moderate = 2, or high = 3–4).

### Assessment of psychometric properties

*Construct validity* was determined by comparing the first administration of the five KOOS subscales against the five WOMAC subscales, the eight SF-36 subscales, and the six Pain-NRS subscales. On the assumption that the highest correlations can be expected to be observed when comparing scales that are intended to measure the same or similar constructs, we posited a priori sets of hypotheses about convergent relationships between physical health properties which are given in Table 4 in the results section. We defined the construct validity of the KOOS questionnaire as good if at least 75% of the hypotheses could be supported [26]. The *internal consistency* of the first-administered KOOS was determined by defining the degree of inter-relatedness among the items. *Test-retest reliability* was determined by comparing the scores of the first- and second-administered KOOS questionnaires. *Measurement error* is the systematic and random error of a patient’s score that is not attributed to true changes in the construct to be measured [27]. The standard error of measurement (SEM) is a measure of the absolute measurement error of how much measured test scores tend to be distributed around a “true” score. SEM is expressed in the unit of measurement of the instrument. The minimal detectable change (MDC), in turn, is the threshold for determining clinical changes outside measurement error. The *floor and ceiling effect* of the KOOS was also examined. We checked each questionnaire for missing values prior to further analysis. The KOOS Users Guide 2012 rule was applied for missing items [21].

### Statistical analyses

Quantitative variables are described using mean and standard deviation (SD) or median and interquartile range (IQR) values, and categorical variables are described using frequency and percentage values. Construct validity was calculated by using Spearman’s correlation coefficient to assess the relationships between the KOOS subscales and the WOMAC, SF-36 and numeric pain rating scales. Correlation coefficients less than 0.2 were considered very weak, between 0.2 and 0.39 weak, between 0.4 and 0.59 moderate, between 0.6 and 0.79 strong, and above 0.79 very strong [28]. Internal consistency was determined by calculating Cronbach’s α coefficient. A Cronbach’s α equal to, or greater than, 0.70 is generally regarded as acceptable for internal consistency [29, 30]. Test-retest reliability, indicating the consistency of the KOOS scores between the first and second administration of the questionnaire, was calculated using the two-way random effect model of the intraclass correlation coefficient (ICC) presented with 95% confidence intervals (CI). ICC values above 0.81 were interpreted as excellent, whereas values between 0.80 and 0.61 indicated good, values between 0.60 and 0.41 moderate, values between 0.40 and 0.21 fair, and values below 0.20 low reliability [31]. SEM was calculated using the formula: SEM = 1.96√(1-R), where 1.96 derives from the 0.95% CI and R represents the calculated ICC coefficient. MDC was calculated using the formula: MDC = SEM*1.96√2, where 2 represents two measurements evaluating the change [32]. For group comparison the MDC can be calculated, depending on the size of the group (*n* = 59), as follows: MDC_group_ = MDC_individual_/√n [33]. The floor and ceiling values representing the percentages of the patients who obtained the lowest or highest scores were calculated for each KOOS subscale separately. Floor and ceiling effects are considered present if more than 15% of the respondents achieve the lowest or highest possible scores [26].

## Results

### Patients

A total of 59 patients were included in the validity, internal consistency, test-retest, measurement error and floor/ceiling effects assessments. Mean participant age was 49 (SD 14), most were women (78%), and the median duration of knee symptoms was 7 months for women (IQR 4, 12) and 6 months for men (IQR 3, 18). Mean self-reported knee pain for all patients was 5.6 (SD 2.6) on a NRS scale of 0 to 10. The characteristics of the study population are presented in Table 1.

**Table 1:** Demographic and clinical characteristics of patients

Variables	Values
Women, n (%)	46 (78)
Age, mean (SD)	49 (14)
Body mass index, mean (SD)	26.5 (4.9)
≥30, n (%)	13 (22)
Employment status, n (%)	
Employed	41 (69)
Unemployed	8 (14)
Retired	10 (17)
White collar workers, n (%)	28 (47)
Duration of symptoms, month, median (IQR)	6 (3, 12)
Onset of knee pain, n (%)	
Accidentally	27 (46)
No clear event	32 (54)
Leisure time physical activity, n (%)	
Low	8 (14)
Moderate	27 (46)
High	24 (40)
Numeric Pain Rating Scale (0 to 10 scale)^a^, mean (SD)	
General	4.5 (2.3)
Neck	2.6 (2.5)
Back	2.4 (2.5)
Upper limb	1.9 (2.6)
Lower limb	3.9 (3.1)
Knee	5.6 (2.6)

*SD* Standard Deviation, *IQR* Interquartile Range

^a^negative number indicates less pain

Only a few individual items (2%), all from the Pain and Sport and Recreation Function subscales, were missing, and hence a total score for all the subscales was available for all patients (Table 2). The lowest possible scores were reported by 5 (9%) patients for the subscale Sport and Recreation Function and by 4 (7%) patients for the subscale QOL. The best possible scores were reported by one patient (2%) for the subscales Pain and QOL, and by 2 patients (3%) for the subscales ADL and Sport and Recreation Function (Table 2).

**Table 2:** Mean scores, response rates and floor and ceiling percentages for the Finnish KOOS subscales

	Mean (SD)	Response rate (%)	Floor^a^ (%)	Ceiling^b^ (%)
Pain	55.4 (22.3)	98	0	2
Symptoms	53.3 (13.4)	100	0	0
Activities of Daily Living	67.4 (23.0)	100	0	3
Sport and Recreation Function	32.9 (25.9)	98	9	3
Knee-related Quality of Life	32.3 (22.5)	100	7	2

100 = no knee problems, 0 = extreme knee problems

*KOOS* Knee Injury and Osteoarthritis Outcome Score, *SD* Standard Deviation

^a^Worst possible value on the subscale

^b^Best possible value on the subscale

### Cross-cultural adaptation

The cross-cultural adaptation revealed minor cultural differences. In the subscale Activities of Daily Living, items A9 and A11 (“Putting on socks/stockings”, and “Taking off stocks /stockings”) the word “stockings” was omitted. In addition, item A13 on the same subscale (“Getting in/out of the bath”) the word “shower” was added as an alternative to bath. The Finnish version of the KOOS questionnaire was well received by the sample of 16 postmenopausal women with mild knee OA. All the questions and response options were considered understandable and applicable. The back-translation of the Finnish version of the KOOS questionnaire is available in the additional file [see Additional file 1].

### Construct validity

Overall, the highest correlations were found between the KOOS subscales and WOMAC subscales. As shown in Table 3, all the KOOS and WOMAC subscales correlated significantly, with values of r within the range 0.33 to 0.86. The highest correlations were between the subscales intended to measure similar constructs (KOOS ADL vs. WOMAC Physical function, *r* = 0.86; KOOS Pain vs. WOMAC Pain, *r* = 0.81). In addition, the KOOS Symptoms and WOMAC Stiffness subscales correlated moderately (*r* = 0.48). Thus, the set of a priori hypotheses posited for KOOS and WOMAC was supported (Table 4).

**Table 3:** Spearman’s correlation coefficients for comparisons of the five KOOS subscales with the three WOMAC subscales, eight SF-36 subscales, and the six Pain-NRS subscales

	KOOS Pain	KOOS Symptoms	KOOS ADL	KOOS Sport/Rec	KOOS QOL
WOMAC					
Pain	0.81	0.37	0.65	0.52	0.54
Stiffness	0.60	0.48	0.79	0.62	0.46
Physical function	0.75	0.33	0.86	0.72	0.63
SF-36					
Physical Functioning	0.70	0.43	0.83	0.67	0.66
General Health	0.11	0.25	0.39	0.13	0.10
Vitality	0.28	0.24	0.43	0.18	0.19
Mental Health	0.12	0.15	0.33	0.06	0.08
Role-Physical	0.39	0.34	0.58	0.36	0.30
Role-Emotional	0,41	0.22	0.60	0.29	0.23
Social Functioning	0.42	0.29	0.64	0.41	0.30
Bodily Pain	0.69	0.46	0.77	0.58	0.51
Pain-NRS					
General	0.42	0.23	0.48	0.44	0.53
Back	0.19	0.17	0.37	0.16	0.33
Lower limb	0.37	0.37	0.61	0.41	0.37
Neck	0.09	0.05	0.35	0.07	0.21
Upper limb	0.18	0.39	0.57	0.28	0.33
Knee	0.66	0.46	0.68	0.50	0.53

All correlations over 0.27 were significant at 0.05 level; N = 59

*KOOS* Knee Injury and Osteoarthritis Outcome Score, *ADL* Activities of Daily Living, *Sport/Rec* Sport and Recreation Function, *QOL* Quality of Life, *WOMAC* Western Ontario and McMaster Osteoarthritis Index, *SF-36* Short-Form 36 Health Survey, *Pain-NRS* Numeric Pain Rating Scale

**Table 4:** A priori hypotheses of the expected correlations and realized correlations between the KOOS subscales and the subscales of the WOMAC, SF-36 and Pain-NRS. The correlations are given as Spearman’s correlation coefficient (r) values

Comparative subscales		Expected correlation	Realized correlation
KOOS Pain	WOMAC Pain	*r* = > 0.79 *(very strong)*	*r* = 0.81, supported
KOOS Symptoms	WOMAC Stiffness	*r* = 0.40 to 0.79 *(moderate - strong)*	*r* = 0.48, supported
KOOS ADL	WOMAC Physical function	*r* = > 0.79 *(very strong)*	*r* = 0.86, supported
KOOS Pain	SF-36 Bodily Pain	*r* = 0.60 to 0.79 *(strong)*	*r* = 0.69, supported
KOOS ADL	SF-36 Physical Functioning	*r* = > 0.79 *(very strong)*	*r* = 0.83, supported
KOOS Sport/Rec	SF-36 Physical Functioning	*r* = 0.60 to 0.79 *(strong)*	*r* = 0.67, supported
KOOS Pain	Knee Pain-NRS	*r* = > 0.79 *(very strong)*	*r* = 0.66, not supported
KOOS ADL	Knee Pain-NRS	*r* = 0.60 to 0.79 *(strong)*	*r* = 0.68, supported
KOOS QOL	Knee Pain-NRS	*r* = 0.40 to 0.59 *(moderate)*	*r* = 0.53, supported

*KOOS* Knee Injury and Osteoarthritis Outcome Score, *WOMAC* Western Ontario and McMaster Osteoarthritis Index, *SF-36* Short-Form 36 Health Survey, *Pain-NRS* Numeric Pain Rating Scale, *ADL* Activities of Daily Living, *Sport/Rec* Sport and Recreation Function, *QOL* Quality of Life

Higher correlations were found for the KOOS scales and the SF-36 scales, indicating their high ability to measure physical health, i.e., Physical Functioning, Role-Physical, and Bodily Pain (Table 3). As with WOMAC, the highest correlation between KOOS and SF-36 was found between the subscales intended to measure similar constructs (KOOS Pain vs. SF-36 Bodily Pain, *r* = 0.69; KOOS ADL vs. SF-36 Physical Functioning, *r* = 0.83). The KOOS Sport and Recreation Function and SF-36 Physical Functioning subscales also correlated strongly (*r* = 0.67), thus supporting the set of a priori hypotheses posited for KOOS and SF-36 (Table 4). The correlations between the KOOS subscales and the SF-36 dimensions of General Health, Vitality, Mental Health, Role-Emotional and Social Functioning, were lower, indicating the KOOS’s ability to measure rather physical than mental health.

The highest correlations between the KOOS and Pain-NRS subscales pertaining to different regions of the body were found for the knee area (*r* = 0.46–0.68) and lower limbs (*r* = 0.37–0.61) (Table 3). In addition, overall general pain showed moderate correlations with most of the KOOS subscales. The hypotheses of a strong correlation between KOOS ADL and Pain-NRS Knee (*r* = 0.68), and moderate correlation between KOOS QOL and Pain-NRS Knee (*r* = 0.53) were both supported. However, KOOS Pain and Pain-NRS Knee showed strong correlation (*r* = 0.66) instead of expected very strong correlation (Table 4).

### Reliability

The mean change in the second measurement varied from − 0.7 in the Symptoms subscale to 8.7 in the Sport and Recreation Function subscale (Table 5).

**Table 5:** Difference between first and second measurement, internal consistency, test-retest reliability, and measurement error of the KOOS subscales

	Change from first to second measurement	Internal consistency	Test-retest reliability	Measurement error		
	Mean (95% CI)	Cronbach’s α (95% CI)	ICC (95% CI)	SEM (95% CI)	MDC (95% CI) in individuals	MDC (95% CI) in groups
Pain	2.8 (−0.3 to 5.9)	0.89 (0.84 to 0.94)	0.86 (0.77 to 0.91)	7.7 (5.8 to 9.6)	21.3 (16.5 to 25.1)	2.8 (2.2 to 3.4)
Symptoms	−0.7 (−3.2 to 1.9)	0.79 (0.69 to 0.89)	0.73 (0.59 to 0.83)	6.0 (4.7 to 7.3)	16.6 (13.0 to 20.2)	2.2 (1.7 to 2.6)
Activities of Daily Living	5.1 (1.7 to 8.4)	0.96 (0.95 to 0.98)	0.83 (0.72 to 0.89)	8.7 (6.4 to 11.1)	24.2 (17.7 to 30.7)	3.1 (2.3 to 4.0)
Sport and Recreation Function	8.7 (3.8 to 13.5)	0.88 (0.82 to 0.94)	0.72 (0.57 to 0.82)	12.2 (9.9 to 14.6)	33.8 (27.3 to 40.4)	4.4 (3.6 to 5.3)
Knee-related Quality of Life	4.2 (1.0 to 7.4)	0.90 (0.84 to 0.96)	0.83 (0.73 to 0.89)	8.3 (6.8 to 9.8)	23.1 (19.0 to 27.2)	3.0 (2.5 to 3.5)

*KOOS* Knee Injury and Osteoarthritis Outcome Score, *ICC* Intraclass Correlation Coefficient, *SEM* Standard Error of Measurement, *MDC* Minimal Detectable Change, *CI* Confidence Interval

#### Internal consistency

Cronbach’s α ranged from 0.79 to 0.96, indicating good internal consistency in all the KOOS subscales (Table 5).

#### Test-retest reliability

The test-retest reliability of the KOOS was excellent for the Pain, ADL and Knee-related Quality of Life subscales, with ICCs ranging from 0.83 to 0.86. In the Symptoms and Sport and Recreation Function subscales, reliability was good, with ICCs (95% CIs) of 0.73 (0.59 to 0.83) and 0.72 (0.57 to 0.82), respectively (Table 5).

#### Measurement error

The SEM values ranged from 6.0 to 12.2, with the lowest for the Symptoms subscale and highest for the Sport and Recreation Function subscale (Table 5). Correspondingly, at the individual level the MDC was lowest (16.6) for the Symptoms subscale and highest (33.8) for the Sport and Recreation Function subscale. At the group level, MDC ranged between 2.2 and 4.4 (Table 5).

## Discussion

This study reports on the cross-cultural adaptation and translation of KOOS into Finnish and its reliability, construct validity and floor/ceiling effects in patients with knee injuries and/or OA. The results indicated that the Finnish version of KOOS has good construct validity, and that the questionnaire is a reliable measure of pain, symptoms, activities of daily living, sport and recreation, and quality of life in Finnish-speaking patients with knee injuries of different kinds.

The psychometric properties of the Finnish version of KOOS were in line with those of the original KOOS [13, 34, 35] and with the Persian version of KOOS [14], which studied a group of patients with knee injuries, similar to those in the present study, although not including knee OA. The mean scores for the Sport and Recreation Function and QOL subscales were considerably lower than the scores for the other subscales, as previously reported [12, 15, 16, 34, 36]. The likely reason for these low values, especially for the Sport and Recreation Function, is that knee injury patients tend to avoid risky activities in their daily lives. The present study demonstrated neither floor nor ceiling effects, as the proportion of worst or best possible percentage scores for the KOOS subscale with the highest scores was only 9%. This demonstrates the appropriateness and comprehensiveness of the questionnaire for a patient population with relatively moderate knee pain and other knee injury symptoms.

The construct validity of the KOOS was determined by comparing the KOOS subscales with the subscales of the WOMAC, SF-36 and Pain-NRS. The KOOS subscales are as representative as those of the WOMAC for measuring pain, stiffness and function. It was therefore expected that strong or very strong correlations between the KOOS and WOMAC subscales would be found. However, in interpreting these coefficients it must be acknowledged that all these measurements overlap to a certain extent. The WOMAC Pain items are included in the KOOS subscale of Pain, the WOMAC Stiffness items are included in the KOOS subscale of Symptoms, and the WOMAC Physical Function items are identical to the KOOS ADL items. Thus, in the latter, the subscale response options (VAS vs Likert) are compared rather than the constructs. Due to overlapping subscales between the KOOS and WOMAC, it was essential in this study to compare and correlate the subscales of the KOOS also with other questionnaire’s subscales assessing similar constructs than those of the WOMAC.

We found, somewhat surprisingly, that the KOOS subscale of Symptoms and the WOMAC subscale of Stiffness showed “only” a moderate correlation. This may partially be due to fact, as referred to above, that the KOOS Symptoms subscale contains five items in addition to the two original WOMAC items, as it also takes symptoms related to knee movement into account. Also noteworthy, alongside the KOOS subscales of Pain, Symptoms and Activities of Daily Living, were the subscales of Sport and Recreation Function and QOL, which showed a moderate or strong correlation with all the WOMAC subscales.

When the KOOS and SF-36 were compared for construct validity, we found strong correlations between the KOOS subscales and those of the SF-36 that measured similar constructs. The highest correlations were observed between the SF-36 subscale of Physical Functioning and the KOOS subscales of ADL and Pain. The SF-36 subscale of Bodily Pain and the KOOS subscales of Pain and ADL also showed strong correlations. In contrast, the KOOS subscale of Symptoms showed the lowest correlations with all the SF-36 subscales. This is in line with the findings of Salavati et al. (2008) in patients with knee injuries, and of Roos et al. (1998) in subjects with knee OA. In fact, Roos et al. concluded that the KOOS Symptoms subscale is not as important as the other four subscales as a determinate of Physical Health. The authors suggested that symptoms and functional limitations should be reported separately and not aggregated into a single score [13]. All in all, the construct validity for the patients in our study was at more or less the same level as observed in patients with knee injuries [14] and less severe forms of OA [18], but higher than that obtained in elderly patients with advanced OA eligible for total joint replacement [15, 34].

The numeric pain rating scale used in this study measures the intensity of pain experienced in general as well as in specific body regions. It contains subscales that make it possible to explore correlations with the KOOS subscales. As expected, we found strong and moderate correlations between all the KOOS and Pain-NRS subscales, particularly those focusing on the lower extremities and the knee region. These findings, in conjunction with the fact that self-reported knee pain was more severe than pain in any other body region, confirm the utility of the KOOS as a lower-extremity PRO measure in subjects with diverse knee problems. Also of note was the finding of strong correlations between the Pain-NRS Knee and lower limb subscales and the KOOS Activities of Daily Living subscale. This is understandable, given that the knee is a large weight-bearing joint with a large range of movements, and that managing the activities of daily living presumes an extensive repertoire of pain-free weight-bearing movements. However, somewhat surprisingly, the KOOS Pain subscale correlated only strongly instead of, as expected, very strongly with the Pain-NRS Knee subscale. This is most likely due to only partial similarity between the constructs in the two subscales. While the nine-item KOOS Pain subscale is designed to elicit the prevalence and degree of pain during different activities and rest, the Pain-NRS Knee subscale consists of a single item in asking about the intensity of pain experienced during the past week. Nevertheless, the use of the Pain-NRS yielded new information, since to our knowledge no previous KOOS validation studies have investigated the correlations between the KOOS and Pain-NRS subscales. From our pre-defined hypotheses altogether 89% could be confirmed.

Internal consistency was good for all five subscales, exceeding the cutoff value of 0.70. This result is comparable to observations in other languages and patient populations, such as in the Swedish version with preoperative patients [13], the Dutch version with patients with focal cartilage defects [37], the Persian version with patients with knee injuries [14], and the Singapore English and Singapore Chinese [15], Dutch [17], Portuguese [18] and French [16] versions with knee OA patients. Two recent Polish validation studies with test-retest intervals of one to 2 weeks found Cronbach’s αs higher than 0.90 in patients undergoing ACL reconstruction [38] and total knee replacement [36]. The authors concluded that this might be due to the relative homogeneity of their patient group. Accordingly, it has been claimed that, for clinical application, high Cronbach’s α values, of at least 0.90, are needed [39]. However, given that, our Cronbach’s α values were, with the exception of the Symptoms subscale, all around borderline significance, we consider them reasonable for clinical purposes. The result of the item analysis also suggests that all five subscales are acceptable for inclusion in the Finnish version of KOOS.

The ICC values for test-retest reliability for all the KOOS subscales were good, ranging from 0.72 to 0.86, thereby indicating sufficient standardization of the KOOS questionnaire. Overall, our results are comparable to those of previous methodological studies of KOOS [12, 13] and other studies [14, 17–19, 36, 37, 40, 41] where ICC values over 0.70 have indicated good or excellent test-retest reliability. Noteworthy, the ICC value for Sport and Recreation Function in our study was somewhat lower than expected. It is commonly seen that Sport and Recreation Function has higher ICC value than Symptoms [17, 36, 40, 41].We cannot find an exact reason why Sport and Recreation Function had relatively low value, but it is possible that some patients may have changed their activity level during 2 week interval.

The MDC value of 4.4 points at the group level indicates that the Finnish version of the KOOS has an ability to detect a minimum change of 4.5 points between the measurements. The MDC should be smaller than the minimal important change (MIC), which is regarded as the smallest change score needed for the effect to be considered clinically relevant [42]. For the different KOOS subscales a MIC of 8–10 points has been considered to be appropriate [34]. Thus the Finnish version of the KOOS is applicable to detect such a change without difficulties. However, it is important to note that the mentioned MDC values apply to patient groups and not to individual patients. The MDC values at the individual level were considerably higher ranging from 16.6 to 33.8 for the different KOOS subscales. The MDC values in our study are of same magnitude or somewhat higher than those found in two recent studies in people with OA awaiting arthroplasty [36, 43].

We recognize some limitations of this study. First, our sample size was rather small. The response rate for the re-test remained low (~ 45%), meaning that a relatively small number of participants were investigated. The reasons why some subjects did not respond to the second KOOS questionnaire remain unknown. Nevertheless, according to the Consensus-based Standards for the selection of health status Measurement Instruments (COSMIN) group [44], the sample size of 50 to 99 is considered sufficient. Second, participants in the present study may not represent patients with entire spectrum of knee OA and some common knee complaints affecting pain, such as patellofemoral pain syndrome, rheumatoid arthritis, plica syndrome, Bakers cyst and bursitis. However, it must be borne in mind that the KOOS questionnaire is intended to be used particularly for knee injuries that can result for a variety of reasons, including OA. In addition, the initial participant group recruited to this study was representative of their population, as all knee patients attending the outpatient clinic were invited to take part to the study. Third, due to its cross-sectional design, the responsiveness of the questionnaire was not assessed in this study, which may limit the instrument’s ability to detect clinically important changes over time. For these reasons, we recommend further validation of this preliminary Finnish version of KOOS and consideration of its responsiveness with a larger number of patients with knee complaints, including patients across the full radiographic spectrum of knee OA.

## Conclusions

This research effort produced an appropriately translated and culturally adapted version of KOOS. The Finnish version of KOOS is a reliable and valid measure that can be applied as a self-report and disease-specific questionnaire for use in middle-aged patients with knee injuries. The responsiveness of the Finnish version of KOOS in larger groups of patients with knee complaints remains to be tested.

## Additional file

Additional file 1: The back-translation of the Finnish version of the KOOS questionnaire “KOOS KNEE QUESTIONNAIRE”. (PDF 87 kb)

## Abbreviations

ACL: Anterior cruciate ligament
ADL: Activities of daily living
CI: Confidence interval
COSMIN: Consensus-based standards for the election of health status measurement instruments
ICC: Intraclass correlation coefficient
IQR: Interquartile range
KOOS: Knee Injury and Osteoarthritis Outcome Score
LTPA: Leisure time physical activity
MDC: Minimal detectable change
MIC: Minimal important change
OA: Osteoarthritis
Pain-NRS: Pain numeric rating scale
PRO: Patient-reported outcome
QOL: Quality of life
SD: Standard deviation
SEM: Standard error of measurement
SF-36: Short-Form 36 Health Survey
VAS: Visual Analog Scale
WOMAC: Western Ontario and McMaster Osteoarthritis Index
