# Supplementary material for: Construct validity and reliability of the Finnish version of the Knee Injury and Osteoarthritis Outcome Score
Source: BMC Musculoskelet Disord. 2018 May 22;19:155. doi: 10.1186/s12891-018-2078-7 (PMC5964707; doi:10.1186/s12891-018-2078-7)
Supplement: Supplementary file 1 — The back-translation of the Finnish version of the KOOS questionnaire “KOOS KNEE QUESTIONNAIRE”. (PDF 87 kb) [file 12891_2018_2078_MOESM1_ESM.pdf]

KOOS KNEE QUESTIONNAIRE
(Back-translation of the Finnish version of the KOOS questionnaire)

Today's date: ______________________ Date of birth: _______________________

Name: ____

INSTRUCTIONS: This form contains questions about how you experience the situation regarding your knee. This information will help us monitor the condition of your knee and how well you are able to cope in your everyday activities.
Answer each question by ticking just one response option. Tick the option that best corresponds to your situation.

Symptoms

Answer the following questions considering the knee symptoms you have experienced during the past week:

S1. Do you experience swelling/inflammation of the knee?

Never	Rarely	Sometimes	Often	Constantly
☐	☐	☐	☐	☐

S2. Do you feel a cracking sensation, hear popping or other kinds of sounds when moving your knee?

Never	Rarely	Sometimes	Often	Constantly
☐	☐	☐	☐	☐

S3. Does your knee jam or lock when you are walking?

Never	Rarely	Sometimes	Often	Constantly
☐	☐	☐	☐	☐

S4. Can you straighten your knee all the way?

Always	Often	Sometimes	Rarely	Never
☐	☐	☐	☐	☐

S5. Can you bend your knee all the way?

Always	Often	Sometimes	Rarely	Never
☐	☐	☐	☐	☐

Stiffness

The following questions concern stiffness in your knee during the past week. Stiffness means experiencing restricted or slow movement of the knee joint.

S6. How stiff is your knee after waking up in the morning?

Not at all	A little stiff	Fairly stiff	Very stiff	Extremely stiff
☐	☐	☐	☐	☐

S7. How stiff is your knee after sitting, lying down or resting during the day?

Not at all	A little stiff	Fairly stiff	Very stiff	Extremely stiff
☐	☐	☐	☐	☐

Pain

P1. How often do you experience pain in your knee?

Never	About once a month	About once a week	Daily	Continuously
☐	☐	☐	☐	☐

To what extent have you felt knee pain when performing the following actions during the past week?

P2. Turning or twisting the knee

No pain at all	Slightly	Moderately	Strongly	Very strongly
☐	☐	☐	☐	☐

P3. When straightening the knee all the way

No pain at all	Slightly	Moderately	Strongly	Very strongly
☐	☐	☐	☐	☐

P4. When bending the knee all the way

No pain at all	Slightly	Moderately	Strongly	Very strongly
☐	☐	☐	☐	☐

P5. When walking on a level surface

No pain at all	Slightly	Moderately	Strongly	Very strongly
☐	☐	☐	☐	☐

P6. When going up or down stairs

No pain at all	Slightly	Moderately	Strongly	Very strongly
☐	☐	☐	☐	☐

P7. When in bed at night (pain interferes with sleep)

No pain at all	Slightly	Moderately	Strongly	Very strongly
☐	☐	☐	☐	☐

P8. When sitting or lying down

No pain at all	Slightly	Moderately	Strongly	Very strongly
☐	☐	☐	☐	☐

P9. When standing

No pain at all	Slightly	Moderately	Strongly	Very strongly
☐	☐	☐	☐	☐

Ability to perform daily activities

The following questions concern your ability to perform physical activities, meaning moving about and looking after yourself. Mark the option that best describes the extent to which the condition of your knee has affected your ability to perform the following activities during the past week.

A1. Descending stairs

Not at all	A little	Moderately	A lot	Very much
☐	☐	☐	☐	☐

A2. Ascending stairs

Not at all	A little	Moderately	A lot	Very much
☐	☐	☐	☐	☐

A3. Rising from a chair

Not at all	A little	Moderately	A lot	Very much
☐	☐	☐	☐	☐

A4. Standing up

Not at all	A little	Moderately	A lot	Very much
☐	☐	☐	☐	☐

A5. Bending down, for example to pick up something from the floor

Not at all	A little	Moderately	A lot	Very much
☐	☐	☐	☐	☐

A6. Walking on a level surface

Not at all	A little	Moderately	A lot	Very much
☐	☐	☐	☐	☐

A7. Getting into a car/out of a car

Not at all	A little	Moderately	A lot	Very much
☐	☐	☐	☐	☐

A8. Going shopping

Not at all	A little	Moderately	A lot	Very much
☐	☐	☐	☐	☐

A9. Putting on socks

Not at all	A little	Moderately	A lot	Very much
☐	☐	☐	☐	☐

A10. Getting out of bed

Not at all	A little	Moderately	A lot	Very much
☐	☐	☐	☐	☐

A11. Taking off socks

Not at all	A little	Moderately	A lot	Very much
☐	☐	☐	☐	☐

A12. When lying in bed (turning, maintaining position of the knee)

Not at all	A little	Moderately	A lot	Very much
☐	☐	☐	☐	☐

A13. Getting into or out of the bath/shower

Not at all	A little	Moderately	A lot	Very much
☐	☐	☐	☐	☐

A14. When sitting

Not at all	A little	Moderately	A lot	Very much
☐	☐	☐	☐	☐

A15. When sitting on or rising from a pedestal toilet

Not at all	A little	Moderately	A lot	Very much
☐	☐	☐	☐	☐

A16. Doing heavy domestic chores (e.g., snow-clearing, mopping floors)

Not at all	A little	Moderately	A lot	Very much
☐	☐	☐	☐	☐

A17. Doing light domestic chores (e.g., cooking, dusting)

Not at all	A little	Moderately	A lot	Very much
☐	☐	☐	☐	☐

Function, sports and recreational activities

The following questions concern your ability to be physically active. Mark the option that best describes the extent to which the condition of your knee has affected your ability to perform the following activities during the past week.

SP1. Squatting

Not at all	A little	Moderately	A lot	Very much
☐	☐	☐	☐	☐

SP2. Running

Not at all	A little	Moderately	A lot	Very much
☐	☐	☐	☐	☐

SP3. Jumping

Not at all	A little	Moderately	A lot	Very much
☐	☐	☐	☐	☐

SP4. Turning/twisting using the injured knee

Not at all	A little	Moderately	A lot	Very much
☐	☐	☐	☐	☐

SP5. Kneeling

Not at all	A little	Moderately	A lot	Very much
☐	☐	☐	☐	☐

Quality of Life

Q1. How often do you think about your knee problem?

Never	About once a month	About once a week	Daily	Continuosly
☐	☐	☐	☐	☐

Q2. Have you changed your lifestyle to avoid possible knee-damaging activities?

Not at all	A little	Moderately	A lot	Very much
☐	☐	☐	☐	☐

Q3. How many problems are caused by your not being able to fully trust your knee function?

No problems	A few problems	Some problems	Quite a lot of problems	Very many problems
☐	☐	☐	☐	☐

Q4. In general, how much difficulty does your knee cause you?

Not at all	A little	Moderately	A lot	Very much
☐	☐	☐	☐	☐

Thank you very much for answering all the questions on this form.
